# Forests in common: Learning from diversity of community forest arrangements in Europe

**DOI:** 10.1007/s13280-020-01377-x

**Published:** 2020-09-13

**Authors:** Anna Lawrence, Paola Gatto, Nevenka Bogataj, Gun Lidestav

**Affiliations:** 1 Centre for Mountain Studies, Perth College UHI, University of the Highlands and Islands, Perth, PH1 2NX UK; 2 Department TESAF, Territorio e Sistemi Agro-forestali, Università degli Studi di Padova, Agripolis, Viale dell’Università, 16, 35020 Legnaro, PD Italy; 3 Slovenian Institute for Adult Education, Šmartinska 134a, Ljubljana, Slovenia; 4 Department of Forest Resource Management, Swedish University of Agricultural Sciences, 901 83 Umeå, Sweden

**Keywords:** Forest ownership, Grounded inquiry, Multilevel governance, Property rights, Social innovation, Sustainability

## Abstract

Europe has a wealth of community forest arrangements. This paper aims to transcend the diversity of locally specific terms and forms, to highlight the value of considering them inclusively. Building on methods to make sense of diversity, we use reflexive grounded inquiry in fifteen cases in Italy, Scotland, Slovenia and Sweden. Within four dimensions (forest, community, relationships between them, and relationships with wider society), we identify 43 subdimensions to describe them collectively. Our approach shows how European arrangements contribute to wider discourses of collective natural resource management. Both tradition and innovation in Europe inform options for environmental governance. Arrangements challenge the distinction between ‘communities of place’ and ‘communities of interest’, with implications for social and environmental justice. They exemplify multilevel environmental governance through both vertical and horizontal connections. Emerging from long histories of political and environmental pressures, they have a role in enhancing society’s connection with nature and adaptive capacity.

**Electronic supplementary material:**

The online version of this article (10.1007/s13280-020-01377-x) contains supplementary material, which is available to authorised users.

## Introduction

There is a long and rich tradition of communities owning, managing and using forests in Europe (Jeanrenaud 2001; Pemán and De Moor 2013). Research has illustrated the survival or loss of mediaeval commons (Gatto and Bogataj 2015), effects of socialism and post-socialism (Bogataj and Krč 2014; Premrl et al. 2015), social innovation (Ambrose-Oji et al. 2015), recent policy programmes (Lawrence and Ambrose-Oji 2015), new groups forming in response to on-going land reform (Hoffman 2013) and effects on forest management and social equity (Lidestav et al. 2013). However, the overall diversity of European community forest arrangements has featured little in international community forestry literature (Gilmour 2016). Hundreds of scientific papers focus on community forest management in Asia, Africa and North America, many describing community forestry as a state- or donor-imposed agenda, others focusing on indigenous traditions of forest management, others on intermediate, multilayered or innovative arrangements (Ito et al. 2005; Hess 2008; Arts 2014; Cossío et al. 2014). While the modern governance language of ‘participation’ and ‘stakeholders’ is often applied to new examples (e.g. McIlveen and Bradshaw 2009), it is challenging to apply these concepts to institutions that have evolved over centuries, and to develop an analysis that includes both ends of this age spectrum.

The shared legacy of the European wealth of arrangements and experiences is hidden in diversity of language, legal and political systems, and it can be hard to see the implications for new models of natural resource governance. In local languages, forest common property regimes are known by terms including *Agrargemeinschaft, allmänningsskog, besparingsskog, urbar, skupnina, gmajna, zemlja, regole, vicinie, comunità*, rendered in English as ‘forest commons’, ‘community forests’, and a range of other terms (Wald und Holz NRW 2012). There is no consistent distinction between a forest common and a community forest. The international development literature uses the terms interchangeably (Chhatre and Agrawal 2009; Naidu 2011; De Jong 2012; DiGiano et al. 2013), while in Europe the term ‘forest common’ refers to particular models rather than all community forest arrangements (Holmgren et al. 2010; Gatto and Bogataj 2015). Specific terms in local languages can mean different ownership structures in different parts of the same country (van Gils et al. 2014). In the USA and UK, ‘community forestry’ often refers to urban forestry (Hauer et al. 2011; Zheng et al. 2013; Lawrence and Ambrose-Oji 2015), while terms translated into English as ‘community forests’ from local terms in Germany and Switzerland can refer to municipal forests (Walz et al. 2008; Böhnke 2011).

Most community forests are commons in some sense of the word, while ‘forest commons’ are either a specific subset of, or a term used interchangeably with, ‘community forests’ and their institutions. Commons originally referred to land owned by one or more persons, but over which others (‘commoners’) have use rights (Short 2008); the term is also (incompatibly) defined as ‘shared resources in which each stakeholder has an equal interest’ (Hess 2006). The term can be applied to the forest or to the institution (Ostrom 1990; Anderies and Janssen 2016), and these meanings are far from universally agreed. Instead, the English word has often been used as a convenient but imprecise translation for European practices.

This paper aims to transcend the diversity of locally specific terms and forms, to highlight the value of considering community forests and forest commons inclusively.

Europe’s diversity of ownership and rights structures and decision-making processes calls for a different approach, one that is not concerned with definitions and translations but with a grounded inquiry into the scope of arrangements for community groups to manage forests. The authors of this paper explored this challenge through a series of meetings and visits between countries in Europe (see Table 1). We became aware of the need to find language and concepts that enabled an inclusive inquiry, to learn from the wealth of models and experiences, rather than be limited by definitions and typologies.

**Table 1:** Authors’ reactions to different models of community forests from three shared forest visits

Where, when	How the case was presented	Our thoughts
Workshop in Niederdresselndorf, Burbach, Nordrhein-Westfalien, Germany, 2011	A forest owned by a cooperative that manages jointly the property of its 320 members, who own in total 10 895 shares. Parts of the forest land are managed as coppice, where each member can harvest firewood from an assigned parcel, while the other part is high forest and jointly managed with the involvement of professionals	“Is this really a community forest? It is managed by foresters. In the UK we have been emphasising the role of communities in decision-making and control—we rarely see a forester managing a community forest” “How can this be ‘community forest’? Owners have distinct and identified shares. Only management and harvesting is shared. I was expecting common undivided ownership as we have in Italy” “Similar to Slovenian cases of forest management but profit is heavily prioritised, in contrast to the Slovenian situation. When and why has the link between community and forest gone? Who/what is a community?” “This is rather similar to a Swedish Forest Common in terms of objectives and management of the high forest. What differs is the comparatively small size and, that the shareholders have their own specified plot assigned for harvesting their firewood”
Conference at Remscheid, Germany, 2013	An example of a well-managed forest in an industrial area, owned by shareholders who mostly live in urban areas distant from the forest	“Sustainable forest management is upgraded by participation, also of non-local investors. Younger members. But, again, who/what is a community? A dedicated forester links motives with forest but not as in Burbach or Slovenia. Principles are comparable but contexts differ” “The owners of this community forest have never met each other, so are they a community?”
Workshop in Cortina d’Ampezzo, Italy, 2014	An eight-hundred-year old community institution managing its own Alpine, continuous-cover, uneven-aged coniferous forest. The land is owned jointly by the whole community, with no individual shares	“The structure and functioning are similar to Slovenia, size is incomparable. (Joint) shareholders are local inhabitants, roles and rights of non-locals are addressed.” “Very impressive to learn about these forest commons and their ability to adapt and survive for 1000 years being so vital and modern. The care for the internal communication and members influence requires a well-developed management structure and certain business model” “These are community forests! And forest commons! And they have such a long tradition and history of institutional adaptation—how can we compare them with British community woodlands, which are no older than 30 years, and often only a few hectares in size?”

Our thinking is influenced by key issues in the commons and community forestry literature. Recent work uncouples ideas of ownership and governance. For example, Agrawal et al. (2008, p. 1460) assert that ‘effectiveness of forest governance is increasingly independent of formal ownership’, while McDermott and Schreckenberg (2009, p. 158) define ‘community forestry’ not in terms of ownership, but in relation to the exercise of power over decisions about access, use and management of the forest. International work on ‘community forestry’ often recognises that the group who share rights and responsibilities for forest management are not equivalent to the geographical community (van Gils et al. 2014). Property rights theory views rights in natural resources as a bundle, identifying six components (Schlager and Ostrom 1992; Meinzen-Dick et al. 2004): access, withdrawal and exploitation (use rights) and management, exclusion and alienation (control rights). All these factors (ownership, governance, community) are affected by state and market influences, including the power of donors mentioned above, and ownership itself can be economically insignificant where state regulation is strong (Bouriaud et al. 2013).

Context therefore affects what is locally understood as a forest common or community forest. In order to include this wide range of possible models we moved away from definitions and described a loose delineation which enables us to work *inductively* from the range of situations which are labelled as community forests or forest commons, rather than *deductively* from a definition which might not apply in practice. Thus our boundary or delineation includes ‘any community of people with a particular forest which is jointly managed by them’. Collectively, we can refer to these as ‘community forest arrangements’. We use the term ‘community’ to include a range of social groupings that manage a forest, each with internal rules (e.g. membership rules, organisation of rights and duties, and external representation). The group has a relationship with the forest (e.g. management rights, practices, and meanings). It also has a relationship with wider society; the group’s existence is recognised by, and is a subset of, wider society. These four components: the forest, the community, the relationship between community and forest, and the relationship with the outside world, form the starting point for our framework.

Our objective is two-fold: to make sense of the diversity of terminology and forms of community forest arrangements by understanding what characterises them and thereby to identify distinctive characteristics and issues associated with community forestry in Europe which contribute to the wider discourse on collective forest management.

## Materials and Methods

Our challenge was to develop an approach that enabled us to identify a set of shared characteristics of community forest arrangements which describe them adequately across national and disciplinary boundaries. We wanted to find innovative methods which build up understanding of what is included, from within a complex and diverse field. We did so by building on other similar attempts to characterise a diverse field in the social or community forestry sector, using inductive and qualitative approaches. Our approach has some similarity to the systems approach advocated by Bossel (2002) who proposes that complex systems need indicators based on the subsystems. In this case, the forest, the community, the community forest governance and the wider governance scale are each subsystems. It contrasts with other approaches which assess evidence of impact. So while we acknowledge and are informed by the large literature building on the work of Ostrom (2002) which examines common property regimes and identifies indicators linked with ‘success’ of the regime (Poteete and Ostrom 2004; Teitelbaum 2014; Baynes et al. 2015), our approach differs from these in not assessing performance.

Instead, to develop an inclusive description of European community forest arrangements, we needed a more inductive approach. We drew on four studies. Genin et al. (2013) examined 11 cases to develop fifty-eight variables describing rural forests. Glasmeier and Farrigan (2005) examined global understanding of ‘community forestry’ and identified twenty-three meta indicators representing the multiplicity of constructs. Lawrence and Ambrose-Oji (2013) developed a framework for describing community forests in the UK context, based on criteria classified into five ‘key elements’ developed iteratively through deductive logic and indicative experience. Finally, Cheng and Sturtevant (2012) build a framework, inductively derived from case study research and observations, to analyse and understand community-based groups involved in public forest management in the USA. Within these (among other dimensions) they distinguish between three levels of social agency: individuals, the collaborative group itself, and participating or external organisations.

These studies describe the process by which they identified indicators and built up a framework in varying degrees of detail. We identified a need to be explicit about ways to develop a reflexive, collaborative and iterative process. This process enabled us to start from the very different definitions and understandings we each had of community forest arrangements in our own countries, and move through cycles of increased mutual understanding and consensus, to a list of subdimensions that were both necessary and sufficient to describe cases that fit the community forests category in our European experience.

This approach has grown out of traditions of participatory research and adaptive collaborative learning (Guijt 2007) which contrasts a more conventional imposed analysis with an interactive process to co-create meaning (Paudel and Ojha 2007). We understood a need to find shared meaning through a co-creative inductive process, building upwards from shared examples. Consequently, we adopted a deliberative collaborative approach as four scientists from four European countries to jointly examine the question. The deliberative approach is advocated in complex systems, used by sustainability science for transdisciplinary collaboration (Popa et al. 2015), or ‘to uncover the public’s informed, considered and collective view on a normative question’ (Burchardt 2014). Here, we applied it to ourselves, to allow ‘informed, value-based reasoning and collective problem solving’ (Abelson et al. 2013) to jointly work through the question of what is mutually intelligible as community forest arrangements.

As the basis for this inductive process we chose a multiple-case design (Yin 2013). Based on each authors’ familiarity with her own country or region, between three and five cases were selected purposively from each of the four countries of the four authors (15 cases in total). Cases had to be diverse and include examples that were typical, and others that challenged our own and each other’s preconceptions of what a community forest or a forest common is, to help us both find common ground and test the boundaries of our delineation. Some models that we initially considered but rejected because they did not fall within this broad delineation included the following: voluntary groups helping to thin woodlands belonging to environmental NGOs in England (because they do not have a direct relationship with a particular wood, and do not therefore have control over decisions and management) and a forest owner association in Slovenia (because in this case their members each manage their own forest individually not jointly).

Each author wrote a short description of those cases from her own country, using initial draft criteria, based on experience and documentation. Essential information about these case studies is summarised in Table 2. For each dimension of the framework (forest, community, community–forest relationship, community relationship with wider society), we then proposed draft subdimensions, with descriptors for each subdimension, aiming for a set of subdimensions which would collectively be both necessary and sufficient to share our understanding of our case studies. Each researcher tested these draft lists on her own case studies; jointly we used published and grey literature, information from key informants, and presented draft result workshops in Germany and Italy to refine them (Table 1). A set of forty-three subdimensions was finally identified and coded (Table 3a, b).

**Table 2:** Summary of case studies

Name and location Website if available	Establishment year, why and how; area; size; production
Bosco di Mestre (Mestre Woodland), Italy http://www.enti.comune.venezia.it/flex/cm/pages/ServeBLOB.php/L/IT/IDPagina/62	2002. To create urban forest in Venice mainland; incentives to farmers for reforestation; target area: 1200 ha; currently 230 ha; recent dominant objectives are public goods (landscape, ecological corridors)
Associazione Forestale Veneto Orientale (Eastern Veneto Forest Owners’ Association), Italy (now known as Associazione Forestale di Pianura) http://www.afvo.it/	2002. Objective: revitalising production, sustainable management; association among municipalities owning forestland; more than 300 ha in 35 forest estates; recent dominant objectives are timber, firewood and public goods (recreation, landscape)
Comunanza delle Regole d’Ampezzo (Community of Ampezzo forest commons), Italy https://www.regole.it/	Thirteenth century. Objective: community livelihood needs; joining eleven historical individual village commons; collectively owns and manages 16 000 ha of forests and pastures; recent dominant objectives are timber, firewood and public goods (recreation, landscape)
Partecipanza di Trino Vercellese (Trino Vercellese forest common), Italy http://www.comune.trino.vc.it/bosco-della-partecipanza	Thirteenth century. Objective: community livelihood needs; common ownership since beginning; 580 ha of oak forest in urbanised area; recent dominant objectives are firewood and public goods (protected area)
Foresta del Comune di Asiago (Asiago communal forest), Italy http://www.comune.asiago.vi.it	Nineteenth century. Objective: community livelihood needs; originally a closed common, turned into a municipal forest; nearly 6000 ha of forests-pastures land; recent dominant objectives are firewood, mushrooms picking rights for local residents
Agrarna skupnost Cerknica, (Cerknica agrarian common), Slovenia	1860. Objective: community livelihood needs; a closed common abolished after WWII, now re-created; 537 ha of Dinaric forest and planted spruce are naturally regenerated owned jointly by over 200 shareholders and managed now for commercial timber to finish denationalisation procedures, maintenance of biodiversity and recreation facilities
Mestni gozd Celje (Urban Forest, Celje), Slovenia https://www.celje.si/sl/kartica/mestni-gozd	1885. Objectives: a city park 29 ha of mixed forests recently owned by municipality; managed and maintained for education and recreation by State Forestry Service and EU funds
Zveza lastnikov gozdov Slovenije (Slovenian Forest Owners’ Association (FOA)), Slovenia http://www.slovenski-gozdovi.org/o-zvezi/poslanstvo	2001. Objectives: commercial management of over 4000 private small-scale owners associated for common marketing, rights revival and education. Forests are mixed, naturally regenerated
Älvdalens besparingsskog (Älvdalen Forest Common, Sweden) http://www.besparingsskogen.se/alvdalen/	1888. Objectives: to improve forest management, timber production and the livelihood of farmers/community. Originally 54 400 ha, presently 72 400 ha. Forestry and hydroelectric power are main sources of income. Management is carried out by professionals
Vilhelmina Övre Allmänningsskog (Vilhelmina Forest Common), Sweden http://www.vilhelmina-allmanning.se/	1918. Objectives: to improve forest management, timber production and the livelihood of farmers/community. 56 500 ha. Forestry, carried out by staff and contractors, is the core activity. Hunting, fishing and leasing contracts on plots for cabins
Vilhelmina Norra Sameby (Vilhelmina North Reindeer Herding Community), Sweden	1886. One of the 51 reindeer herding communities (RHC) with exclusive right of reindeer husbandry on any forest land Northern Sweden. This RHC organises 30 family enterprises that use about 1.2 million hectares for reindeer grazing
Sala kommunskog (Sala Municipal Forest), Sweden www.sala.se/?page=info&id=12415	1624. Objective: urban fringe forest aiming at timber production, also considering and adapting to nature conservation and recreational use. 5000 ha
Wooplaw Community Woodland, Scottish Borders, Scotland, UK http://www.wooplaw.org.uk/	1985. Objective: managed for education, woodland skills training, recreation and sustainable production. The first woodland bought by a community group in the UK, owned by a community company. 20.3 ha in the Scottish Borders; Increased woodland area; thinning; coppicing
Kilfinan Community Forest, Argyll, Scotland, UK http://www.kilfinancommunityforest.co.uk/	2012 with further forest purchased 2015. Planted by the state forest enterprise, purchased by the community through a community company; 561 ha of commercial conifer plantation in rural western Scotland; objective: managed for commercial timber to support local employment, recreation, skills and training, and affordable housing, and for recreation and biodiversity
Malls Mire Community Woodland, Glasgow, Scotland, UK https://www.urbanroots.org.uk/community-woodland/	2012, leased to an NGO (with local volunteer group) by Glasgow City Council (owner); 9 ha; a small mixed urban wood on former industrial wasteland created in 2008; objective: managed for biodiversity and quality of life; ownership by municipality prohibits commercial production

**Table 3a:** Description of the four dimensions

Dimension	Description
**1. Forest characteristics**	This dimension analyses the community forest as a physical asset and its role in the wider landscape.
**2. Community characteristics**	This dimension focuses on the characteristics of the community forest group which owns or manages the forest. It considers its structure, processes of formation and membership, and of decision-making. It focuses on the people and their inter-relationships.
**3. Relationship between community and forest**	This dimension analyses the relational aspect of the community forest group with its forest. It focuses on collective action rules and rights, and the signficance of the forest resource for the community.
**4. Relationship between community and wider society**	This dimension analyses the interactions between the community forest group and its assets, and wider society. This is informed particularly by an understanding of multilevel governance and takes into account policies and public agencies influence on the groups and their forests.

**Table 3b:** Framework of dimensions and sub-dimensions for describing community forests models

Dimensions and subdimensions	Descriptors
***1. Forest characteristics***	
1.1 Class of forest size in hectares	< 10; 10–100; 100–1.000; 1.000–10.000; 10.000–20.000; 20.000–100.000; > 100.000
1.2 Productivity of community forest	low; moderate; high
1.3 Share of forest area over the total community area	< 25%; 25–50%; 50–75 %; > 75%
1.4 Changes in the forest in the last ten years	quality unchanged; quality increased; quality decreased; area unchanged; area increased; area decreased; quality and area unchanged; quality and area increased; quality and area decreased
1.5 Importance of forest in broader landscape	not important; moderately important; important
1.6 Urban or rural character	urban; semi-rural; rural, remote; combination
***2. Community characteristics***	
2.1 Community of place or of interest	place; material interest; immaterial interest
2.2 Ease of community identification	no visible signs; some signs, records; easy, clear boundaries, records; difficult to answer
2.3 Legal structure of community	public body; cooperative; association, consortium; company limited by guarantee; charity, foundations; special status; shareholder company; other
2.4 Classes of community size	< than 10 members; 11–30 members; 31–100 members; 101–500 members; 501–1.000 members; > 1.000 members; difficult to answer
2.5 Time of existence of community (age classes)	up to 30 years; 30–100 years; 100–300 years; > 300 years
2.6 Formal regulations on community permanence	none; the community can be dissolved; the community cannot be dissolved; difficult to answer
2.7 Participation in decisions on community functioning	decisions taken outside community; decisions taken by community delegates; decisions taken by all members
2.8 Prevailing model of decision-making	authoritarian; some procedures exist; democratic models followed; democratic models followed/monitored
2.9 Internal conflicts on community functioning	occur, often or sometime unsolved; occur, mostly solved; rare or absent; difficult to answer
2.10 Level of technical knowledge on forest management	all decisions delegated/subcontracted; some decisions delegated/subcontracted; all decisions made by community
2.11 Ease of identifying members of community	not easy; definite membership but poor records; definite membership and clear records
2.12 Ways of acquiring membership	close membership; semi-closed membership; semi-open membership; open membership; not applicable; difficult to answer
2.13 Ways of losing membership	through change of residence; defined by more criteria, rules; complex and monitored; membership is never lost; not applicable
2.14 Sense of attachment to community	weak; medium; strong
2.15 Pro-active engagement of the community members	no attendance, initiatives; low attendance, minimal initiatives; medium attendance, some initiatives; regular attendance, initiatives
***3. Relationship between community and forest***	
3.1 Form of tenure	informal management agreements; formal management agreement; lease; owns without alienation rights; owns with alienation rights; other tenure models
3.2 Rights are connected to	individuals; community forest group; families; land; other
3.3 Rights specific to community members	access, withdrawal/exploitation; access, withdrawal/exploitation, management; access, withdrawal/exploitation, management, exclusion; access, withdrawal/exploitation, management, exclusion, alienation; difficult to judge
3.4 How rights are transferred between individuals	inheritance; purchase of shares; inheritance and/or purchase; other
3.5 Divisibility of rights	joint management but real shares; joint virtual shares, co-ownership; joint indivisible ownership; not applicable
3.6 Importance of forest resource for the community	not important; additional resource; key resource; symbolic; mixed importance
3.7 General objectives of forest management	conservation; income; local development; livelihood, heritage; income, heritage; income, conservation
3.8 Importance of forest production objectives	low; medium; high; not applicable
3.9 Importance of forest livelihood objectives	low; medium; high; not applicable
3.10 Participation in decisions on forest management	decisions taken outside community; decisions taken by community delegates; decisions taken by all members
3.11 Benefits to community members	non timber forest products (NTFP); firewood, other; firewood, timber, NTFP, other; other non-monetary products; money; firewood, money, NTFP, other; firewood, money, NTFP, timber; money, other; no dividends
3.12 Business model	private profit-making business; social enterprise; no business/non-profit; other forms
3.13 How forest uses are monitored	not monitored; monitored by community members ; monitored by public institutions; mixed control
***4. Relationship between community, forest and society***	
4.1 Representativeness of community in statistics	unrepresented; indirectly or inadequately represented; represented
4.2 Society rights on forests, including community forests	access; access and withdrawal; exploitation; management; exclusion; alienation; difficult to answer
4.3 Importance of community forests public goods	none; low; medium; high; difficult to answer
4.4. Scale of community forests public goods	local; regional; national
4.5 Importance of CPR issues on community forests	low; medium; high
4.6 Additional legal constraints on community forests	none; on forest management; on dividends; other
4.7 Support by public institutions to community formation	none; low; medium; high
4.8 Support by public institutions to community functioning	none; low; medium; high
4.9 Involvement of non-members in decision-making	none; informal; formal consultations; formal decision-making; difficult to answer

The question of validity and reliability of such collaborative deliberative approaches is little discussed (Jaramillo et al. 2017), and our approach as a collaboration among researchers (rather than between researchers and their subjects) is innovative. Our experience is that the deliberately wide choice of examples for inclusion, combined with our discursive and iterative approach, plus presentation and validation in two international seminars, makes it highly likely that a similar set of descriptors or indicators would be identified by researchers who were already knowledgeable about community forestry in Europe and internationally. Furthermore, the process and the subdimensions identified provide the basis for insights into variability across Europe, and can reasonably be taken as a working hypothesis for further exploration and testing in other contexts (Yin 2013). Ultimately, the outcome is to make significant progress in clarifying the diverse and confused field of community forestry in Europe.

## Results

A pen picture of a typical community forest arrangement emerging from our subdimensions is as follows. The *forest* is predominantly forest habitat and located in rural/remote areas. The *community* is place-based, has legal status, has relatively large membership, is well established historically and has explicit rules to impede its dissolution; it uses democratic decision-making, and members have a strong sense of attachment to the group and forest. The *community’s relationship with the forest* is one of ownership through jointly held rights, providing livelihood (material) benefits from the forest rather than distributing dividends. The *community’s relationship with wider society* includes the provision of public goods, formal mechanisms for involving local society in decisions, under legal mechanisms set by the state or local public authorities.

There are many variations on this pen picture. In what follows, we describe findings within each of the four major dimensions mentioned above (summarised in Table 3a). Numbers in parenthesis refer to the subdimensions listed in Table 3b (for detailed results showing distribution of descriptors in each subdimension see Appendix S1).

### Forest

We found a wide range of variations on forest size (subdimension 1.1), with no predominance of large or small forests, although the more economically significant were larger. The smaller woodlands were found in the UK and in urban areas of the other countries. In the Alpine region, common property forests are larger than those under other forms of ownership, because they have managed to remain undivided over a long period, while most private forests and municipal forests were cleared in the past centuries. In the Italian Alps, those forests owned by communities are of better quality than others. In Sweden, common forest land is often of poorer quality than private forest land, but of higher quality than state forest land in some regions (Holmgren et al. 2004). In the broader landscape context, most of our cases were ‘important’ (1.6), had more than 75% forest cover (1.3) and were judged to be ‘moderately’ productive (1.2). All had changed over the last decade: most cases were judged to have increased in quality but are stable in terms of forest area (1.4). In terms of location, while urban and remote rural forests predominated, scattered and peri-urban ones were also well-represented (1.6).

### Community

People in communities can be connected through practice and symbolic meaning, as well as place and interest. The majority of communities are based on ‘place’ rather than ‘interest’ (2.1), but the separation of ‘place’ and ‘interest’ as a basis for community can sometimes be unclear, and interest can be ‘material’ (e.g. firewood) or ‘non-material’ (e.g. protecting a valued landscape or traditional practices). We found few groups connected through shared practice; in some traditional forest commons members are rather detached from the forest, their interest consisting more in receiving the benefits than in making decisions. This happens particularly in municipal forests where the mode of decision-making is delegated to local politicians and officers, although citizens can participate.

Some communities are younger than 30 years, while a few are older than three centuries (2.5); their size ranges from ten to more than one thousand members (2.4). Public bodies or associations/consortiums have larger communities, and communities of interest are smaller than communities of place. Communities are usually, but not always, easily identified (2.2) and have clear membership records (2.11). In groups with a long history, or where rights have been interrupted (e.g. by decades of communist government), living heirs may be dispersed around the world and difficult to identify, or inheritance not formally resolved (Premrl et al. 2015). In some cases, numbers of members may not be the same as numbers of shareholders where, for example, membership is held not by individuals but by the farmstead or the family.

We identified a great range of legal structures for community groups (2.3). Legal structures are often specific to individual countries or situations, as with historical models governed by specific laws and by-laws. Conditions under which individuals acquire community membership (2.12) involve complex rules and mechanisms to protect and conserve membership. These include buying or inheriting a property with shares in the “common” (Älvdalen and Vilhelmina); becoming a resident (Sala or Asiago); being of Sami origin and having inherited a “reindeer mark” (for the identification of the ownership of each reindeer (Vilhelmina North Reindeer Herding Community), or belonging to a family originally from the area (Ampezzo, Trino and Cerknica). In some cases, membership is maintained only while members reside in the village, respect the community rules and do not challenge the community ethos through individualistic behaviours (2.13). Formal rules sometime prevent the dissolution of the community (2.6), as in Regole d’Ampezzo, Trino, Vilhelmina and Älvdalen, while in Scotland community ownership often includes an ‘asset lock’ which prevents sale of community land to private individuals.

Most communities follow a democratic model of decision-making, with all members participating in decisions (2.7) either directly or by delegating decision-making power. Engagement may be related to a strong sense of attachment to the community group (2.14). Cases differ in the extent to which members are passive or proactive in attending meetings, making financial contributions and taking initiatives (2.15). In some cases, such as Comunanza delle Regole d’Ampezzo and Cerknica, democratic processes are strictly followed and monitored through charters and by-laws (2.8). In other models, decisions are taken outside the community; for example, in Malls Mire, the local government, which owns the woodland, limits the decisions that the community group can take. Internal conflicts are invariably present and in a few cases were judged as ‘difficult’, while others demonstrate capacity to solve disputes (2.9).

### Relationship between community and its forest

Governance arrangements are diverse, including property rights, decision-making processes and distribution of benefits. Forms of land tenure are heterogeneous (3.1): only five cases include full ‘ownership’ including alienation rights; other models include ownership without alienation rights, leasehold and management agreements with landowners. Restriction of the right to sell did not appear to affect functioning and could protect the common or shared status of the property. Rights in some cases are held by individuals, in others by the community as a whole, by households or as a feature of ownership of particular farms (3.2).

The bundle of rights owned by the individual members of the community varies widely between cases (3.3). At a minimum, members have use rights, and usually also have management rights. In the case of the reindeer herding community, rights are restricted to the maintenance of reindeer husbandry practice, which does not include the management of forest for timber production. Rights can be transferred through change of residence, inheritance, investment or membership (3.4). In ten cases, the community rights are not divisible, being held under either joint indivisible ownership or joint virtual ownership, i.e. individually held shares not connected to specific parcels; in one case, we found joint management of individually held shares (3.5).

In three cases, the forest was considered a key resource for the community (3.6), while in five others it is an additional resource. In some cases, it is a symbolic resource. The symbolic connection can represent the reversal of felt historic injustices (e.g. in Scotland and Slovenia), the survival of something precious (in Italy and Sweden) and the first step towards possibility of a more sustainable lifestyle (Scotland). Multiobjective management was widespread (3.7); forest production was judged moderately or highly important in eleven cases (3.8), as also for livelihood objectives (3.9). The distribution of dividends or benefits from the forest is as ‘money’ in three cases, while most of the remaining cases provide a bundle of benefits including firewood, timber, money and non-wood forest products (3.11).

The range of business models includes profit-making business, ‘social enterprise’ and non-profit (3.12). In five cases, operational decisions are taken by elected or delegated representatives, in four cases directly by members of the community, in three other cases outside the community (3.10). Monitoring of the forest is done by both community members and the public institutions in most cases, less often by either community members or public institutions; in one case, there was no monitoring (3.13).

### Relationship between community and society

Forest commons are poorly represented in official statistics, where they often cannot be distinguished from other types of ownership (4.1). For example, Vilhelmina and Älvdalen Forest Commons were until recently categorised as “other ownership” although in fact privately owned, while the ownership of Regole d’Ampezzo, Partecipanza di Trino and Cerknica is indistinctly reported under the broad category of “other private ownership”. In Scotland, most communities which own forest do so by forming a ‘company limited by guarantee’, so community forests have sometimes appeared as ‘corporate ownership’ in statistics.

Non-members of the community forest group have access rights to the forests in all our cases, and sometimes also withdrawal rights (4.2). In most cases, the community forest provides public ecosystem services (4.3), recognised to varying degrees at a local or regional scale (4.4). Four cases were affected by common-pool resource tensions (4.5): for example, in Vilhelmina North Reindeer Herding Community, the grazing resource for reindeer is severely affected by other land use activities such as forestry, mining, and hydropower and infrastructure (Sandström 2015). It is striking how relationships with others are tied up with the identity of the community forest group: for example, if the community has exclusion rights, this may reduce the impact of the public on the forest and avoid tragedies of the commons. However, in several cases, decisions about the forest include others beyond the community (4.9), through legal constraints on distribution of dividends (4.6) in six cases. On the other hand, communities receive support and protection for their functioning from local authorities and public institutions (4.7 and 4.8): for example, a law in Italy recognises the distinctive role of commons as key actors in local development and in conservation of environmental and cultural heritage.

## Discussion

Our research aimed to make sense of the diversity of terminology and forms of community forest arrangements by understanding what characterises them and hence to identify distinctive issues associated with community forestry in Europe. In this section, we summarise the contribution made by our framework before considering particular issues which characterise the forests, the communities, the community forest governance arrangements and the wider societal arrangements.

### Characterising community forest arrangements in Europe

We started with a loose indication of community forest arrangements which helps to achieve inclusivity and to focus on the *relationship* between an actual forest and an actual community situated in wider society. Within these limits, we included 15 models from our own four countries, no two the same. Some have only recently come into being, others have survived and adapted over nearly 1000 years. By focusing on the commonalities, we highlight a resource for communities and support organisations, and entry points for further research into forest governance and the relationships between context, model and outcome. In an overview in the USA, researchers concluded that community forestry is defined only implicitly and rather superficially, such that it ‘falls short of universal characterisation’ (Glasmeier and Farrigan 2005). Europe presents an even more diverse situation, but by setting boundaries that focus on the relationship between community and forest, we find that it can be usefully characterised. Like Glasmeier and Farrigan (2005), the understanding of community forestry or forest commons is time and place dependent. However, in Europe, the multitude of models for community forest arrangements has evolved without reference to a unifying concept; they vary in terms of both ideal form and the real details of implementation. Our framework thus both highlights the value of bringing these models together across these four very distinct European countries and enables us to identify the many variations on the concept.

The four main dimensions of the framework form the basic structure of our delineation and provide a logical structure for analysis. Within that, our inductive and reflective approach shows that the forest resource itself is highly variable. The subdimensions identified for ‘community’ and ‘community–forest relationship’ help to distinguish between the internal rules of who belongs to the group and shapes decisions, and the management and use of the forest. The fourth dimension draws attention to how the community forest arrangements are legitimised and supported by law, policy and society.

The methodology has internal validity based on the repeated scrutiny of meanings and mutual understandings, and external validity because the resulting framework works in describing community forest arrangements in our four countries. It provides a set of concepts and questions for describing or interrogating how the community works, how it manages its forest and how external relations shape that. As with comparable methods the framework is a heuristic tool (Cheng and Sturtevant 2012). It is not set in stone but the iterative testing of criteria suggests that it is complete enough to be useful for groups to analyse their own arrangements and compare with others, for communities to ask questions about membership, participation and sharing of benefits, and for researchers to further explore the wealth of models in Europe and beyond.

### The forest resource

The forest resources owned and managed by communities in the four countries illustrate a wide range of possibilities. Of the four dimensions, this one is the least amenable to generalisation. It seems that potentially any forest can be a community forest. This finding is strengthened by the fact that we selected our sample based on community governance criteria, not on the type of forest governed. Because the framework includes the size and condition of the forest, our findings highlight some issues for further research in community-managed forests.

The first is the condition and production potential of the forests. Some of the examples include valuable economic resources, with potential or actual contribution to household and local economies (Lidestav et al. 2017). This contrasts with a situation often highlighted in the international literature, which draws attention to the sometimes poorer quality of forests offered to communities for management (Gibson et al. 2005). It might be hypothesised that the poorest quality community forests that are ‘handed over’ to communities result from central policy decisions to transfer tenure of less valuable forest. The diversity within Europe provides material to test and challenge this narrative.

The second is the inclusion of urban forests. In all four countries our delineation led us to include forests managed by local governments and NGOs on behalf of a geographically and politically defined community. In these, the quality of the resource is judged more according to amenity, well-being and nature conservation (e.g. Draper 2001) and community governance is linked to socio-ecological criteria (Gulsrud et al. 2018). Our cases illustrate innovations aiming to produce (at least some) timber and firewood, within urban areas. This combination of urban, community governance and forest production merits greater attention in sustainable development.

Third was the finding that all the chosen examples were judged to have improved in quality. We did not aim to assess outcomes of community forest arrangements, and this finding does not claim a causal relationship between tenure and forest quality (see Conclusions for further reflection on this issue). However, this assessment suggests that it would be valuable to research the changes in forest condition and value as perceived or measured by various stakeholders over time. This would provide insights into what is valued in different community forests and, if both quantitative and qualitative indicators were included, could be of value in raising the profile of community forest arrangements.

### Community and context

By focusing on the community separately from the community’s engagement with the forest, we highlighted issues around rules for inclusion, where the European experience has a particular contribution to make. Community forest arrangements in old and new Europe challenge the boundaries between ‘communities of place’ and ‘communities of interest’. Researchers globally have reflected on increasing plurality and mobility of communities, leading to more multiscalar and delocalised communities (Nelson and Pettit 2004; Ojha et al. 2016). Harrington et al. (2008), rejecting an ‘excessive focus on place-based communities’, interpret this as a need to pay greater attention to ‘who *should* be involved’ [our emphasis] and how.

This critique builds on, and goes beyond, earlier critiques of ‘participatory exclusion’ (Agarwal 2001), the idea that externally imposed notions of participation can reinforce elite dominance, or existing gender or ethnic biases. Instead, it implies that certain groups merit intentional inclusion, and they are not always those most physically present in or near the forest. It is important to distinguish, however, between these discourses about participation and the stricter definition of community management that we refer to here. Following Charnley and Poe (2007) and many others, our delineation focuses on communities in *control* of the management decisions, i.e. not those who simply have a right to voice an opinion or participate in some way, but rather those who own, or have significant management powers in, their forest.

The European context offers something additional to this critique. Our examples from four countries provide a wide range of models of who *is* involved, and what the existing rules of inclusion and decision-making are. The group of people who share rights to the forest is usually well defined, but may be place- or ancestry-based, may or may not map on to the whole place community and may or may not be accessible to newcomers and distribute equal benefits. Many of these models have emerged and evolved; unlike policy-led models in much of the rest of the world, they have usually not been designed. This provides a natural experiment in forest governance, which is a rich basis for future research.

It also provides an opportunity to investigate questions of environmental justice. Debates about equity in community forestry recognise differences among social groups in terms of decision-making power and benefit distribution (McDermott and Schreckenberg 2009). European modes of shared forest governance highlight additional issues for equity, meaning and place attachment. In some of the older forest commons, shares are inherited, so that different households in a village may have different rights to benefit from the forest. Gender equality is challenged by traditions which pass forest shares to the males of the next generation (Lidestav 2010; Casari and Lisciandra 2016). Rights between different ethnic groups are contested and negotiated in the reindeer grazing grounds of the far north (Widmark and Sandstrom 2012). Different land reform legislation applies to different areas of Scotland (Brown 2008). The intersection of geography, history, personal and cultural meanings for the forest does not sit easily with a monolithic understanding of equity, and sustainable governance will have to engage with the fluidity of this arena. Future adaptation of these models may require critical reflection on the interface between tradition and environmental justice, informed by both European history and reactions to donor- and policy-led interventions typical of other parts of the world.

One other way in which our inclusive approach challenges convention is in the treatment of municipal or local government forests. ‘Municipal forests’ are a longstanding institution in many countries of Europe, where towns or communities own forests through their local government. These are often labelled ‘communal forests’ rather than ‘community forests’ or ‘forest commons’ but the terminology is not applied consistently; furthermore, they are sometimes treated as public, sometimes as private, forests (Weiss et al. 2019). In some, such as the communes of southern France and Switzerland, the connection between community and local government is strong, and residents can be seen as participating directly in forest management (Finger-Stich 2005). These forests fit our broad delineation of ‘any community of people with a particular forest which is jointly managed by them’. In others, including some of our cases, the management is delegated to a local government agency or a non-profit organisation, on behalf of the community, but there is still a direct connection between the community and the forest. Again variations on this theme across Europe may have much to contribute, for example by providing a more equitable route to benefit sharing from public forests.

### Managing forests in common

It is likely, but not consistently demonstrated, that communities manage forests in a different way from other kinds of owners. Why might we expect this? The perception that forest quality has increased might point towards good silvicultural standards; alternatively, the benefits required from communally managed forests might be different from those of state, commercially or individually owned forests and require different management approaches. For example, forest commons in Romania are an important source of domestic firewood (Hartel et al. 2014), while forest commons in northern Spain have high biodiversity value owing to low management intensity (Guadilla-Sáez et al. 2019).

Ostrom (2012) cautioned against recommending ‘optimal’ solutions for management of common-pool resources, and advocated experimentation to find out what worked in what conditions. By observing and comparing diversity we have a form of virtual experimentation. Of our 15 cases, although each was ‘normal’ in its context, no two presented the same combination of property rights, silvicultural and harvesting practices. Some European models have survived and adapted over 1000 years, others were imposed in the nineteenth century, and others result from innovation in the last 20 years. Europe thus provides an opportunity to explore deep-rooted historical influences on arrangements (Moen and Keskitalo 2010; Bouriaud et al. 2015). For example, similar models in Slovenia and Italy had very different experiences in the twentieth century (Gatto and Bogataj 2015). Even between neighbouring former socialist countries such as Latvia and Lithuania, very different trajectories are followed (Brukas 2015).

In addition to historical and political contingency, diverse forest management systems are also derived from social innovation, i.e. from the rearrangements of decision-making power among the communities in question (Moulaert et al. 2013). We see novel modes of community forestry across Europe, including community involvement in urban forestry in Italy and the Netherlands, which led to more landscape scale management (Buijs et al. 2018); associations of individual owners to enhance forest management in Portugal which open possibilities for more sustainable management (Martins and Borges 2007; Carvalho-Ribeiro et al. 2010), and opportunities for urban dwellers to buy shares in new forest commons in Germany (Wald und Holz NRW 2012).

Both novel arrangements and renewed attention to more traditional community forest arrangements create a change in involvement of communities, and opportunities for more meaningful relationships between community members and the forest. ‘Meaningfulness’ or relational values of forest commons and community forests are rarely taken into consideration in contemporary studies of community forestry, but a host of recent studies show that relational values are central to environmental motivations and management of social–ecological systems (Arias-Arévalo et al. 2017). Hajjar et al. (2014) have also argued that meaningful and participatory dialogue on strategies for forest management requires participants to understand forest management. Joint management and the need to consider the forest and make decisions about its management are likely to enhance this understanding.

### Relating to wider levels of governance

The fourth dimension of our framework, relationship between community and society, is an important one because forest commons do not exist in isolation. They are validated, delegitimised or promoted, by culture, legislation and prevailing economic philosophies (Paletto et al. 2013). In Scotland, although the initial urge towards community ownership came from grassroots movements (Ritchie and Haggith 2005), a series of laws from 2003 onwards has created new legal forms and opportunities for community forests (Bryden and Geisler 2007; Mc Morran et al. 2018). In Sweden, what appear to outsiders as long-established traditions are in fact the result of government programmes in the nineteenth century (Holmgren et al. 2010). In Slovenia, the centralising and nationalising effects of socialism disrupted a 1000-year-old model of forest commons (Gatto and Bogataj 2015).

Numerous examples across Europe illustrate the wider relevance of this point. In Portugal, traditional commons known as *baldios* were afforested by the State, but returned to local government ownership in 1974, with mixed results (Skulska et al. 2020). In Spain, by the late eighteenth century, politics did not recognise community ownership as a form of property, and forest commons were privatised with resulting loss of forest (Guadilla-Sáez et al. 2020). While ancient modes of common forest management persist, and new ones emerge across Europe, in places they are also neglected or overlooked. Private charters have replaced informal arrangements in the pastures and forests of the Italian Alps (Casari 2007) while in Switzerland, mediaeval arrangements are under stress (Kissling-Näf et al. 2002). In Sardinia, appreciation of common rights has declined, and common lands are now managed by municipalities (Paletto et al. 2013).

This variable official status of common forests is reflected in mixed and generally poor representation in national statistics. In Scotland, one set of government statistics monitors land owned by communities, while another completely separate process collects very broad data on forest ownership, in only two categories—private and public (Forest Research 2019; Scottish Government 2019). A new inter-government study of forest ownership in Europe and North America finds it impossible to summarise community ownership across the different countries; some is recorded as public, other as private; some can be seen as a ‘third type’ between public and private (UNECE/FAO 2019).

Both the findings that there are abundant thriving models of community forestry and that they are ambiguously legible in national statistics reflect growing attention to multiscale governance (Ostrom 2007). Overlapping scales, horizontal and vertical connections provide abundant scope for social learning and co-adaptation (Gretter et al. 2018). Where community forest arrangements are accommodated, supported or even delivered by government or other regional bodies, there is a formal structure to the multiple scales, more often studied in the global South than Europe (Cronkleton et al. 2011). The variability of public institution involvement in forming, or supporting, community forest groups in our cases highlights the complexity of relationships between state, private and civil society actors, and cross-linkages at wider scales including global (Mwangi and Wardell 2012). Donor-funded intervention in European community forestry has been less significant than in other continents (cf. Rahman et al. 2016), but international funding has been influential in parts of Europe. Neoliberal programmes to support forest owner associations are driven by the World Bank in parts of post-socialist Europe and are variously viewed as contributing to improved management and outcomes, a ‘new imperialism’ and/or privatisation of community resources (Fagan 2006; Sandulescu et al. 2007; Burns et al. 2017).

## Conclusions

Our analysis of community forest arrangements in four European countries highlights diversity of approach to a common challenge, i.e. how to share power, knowledge and benefits through management of forests. By moving away from legalistic and normative approaches focusing on ideals, forms and definitions, and focusing instead on community forest arrangements as encountered in contemporary reality, we highlight a class of social-ecological systems that transcends local specificities. We have demonstrated a variety of arrangements including ancient or new, celebrated or suppressed, flourishing or abandoned, and we have provided numerous examples to show that this diversity extends across the continent. That so many examples are thriving and innovating shows their value for contemporary societies. Yet each arose in local context and (mostly) without awareness of conceptual categories such as common property regimes, community forest arrangements or forest commons.

The wealth of community forest arrangements in Europe provides fertile ground for innovation in forest governance, but this has been inhibited by poor visibility and by differences in language and interpretation, both within Europe and when comparing other continents. Many cases are not recorded in official statistics or are recorded ambiguously or inaccurately. Visibility helps not only to avoid injustices, but also to strengthen the potential for these forms to offer models for sustainable resource management and human ecology.

There is of course a risk of circular argument here, because the reason that many forms of community forest arrangements are not recorded is that they are not seen as part of the broader category of social–ecological system that we are promoting. Perhaps there will never be incentives for individual countries to gather official statistics on this, but greater visibility to the wider category—forests managed collectively by a defined group or ‘community’ of people—would be most valuable in drawing attention to their value.

The core purpose of this paper is to consider this category of arrangements as a whole. Only by so doing will we see ways to innovate, question fairness of process and outcomes, explore wider generalisations about property and its effect on ecosystem services and human values for nature and develop the policies, intermediary organisations and other features of an enabling environment that support common forests to be part of a sustainable future. Only by considering them as a whole can we see the value of tradition and innovation in Europe compared with other continents. History provides us with a suite of options that fall outside normal design parameters, while novel models demonstrate social innovation in its most useful sense as a radical reconfiguration of social relations. European variations on community, eligibility and justice enliven international discourse with some surprising, some inspiring and some questionable approaches.

Our paper does not focus on outcomes. Our task was to investigate both diversity and conceptual unity of the community–forest interaction when that arrangement involves governance and forest management. We have demonstrated unifying themes and provided a spectrum of different forms. These when extended more widely across Europe provide a rich resource for future research of outcomes and adaptation. There is of course a sense that the models we included are in some way successful, in that they exist and in some cases have continued to exist for hundreds of years. But their contemporary relevance is in many cases very different from the subsistence-livelihood context in which many were established. Even the newer models, such as urban forests in Italy or land reform in Scotland, provide experiences unimagined by those who planned them. Relationship is an inextricable component of community forestry, and humans engaging consciously with forests will be changed by the experience.

For these reasons, we commend this field as one with great potential for further study of outcomes, beyond the simplistic binary of ‘success’ or ‘failure’. Evaluative approaches can be narrowly concerned with success, often based on measures of production. Prevailing power structures beyond the community, including central government and international funding influences, want to assess community ownership, social innovation and social enterprise on the basis of economic measures but we suggest—based on the case studies in our four countries and wider reference to many others—that existence, survival, healthy functioning and meaningfulness of the forest governance model are as important as impact. That which is valued has meaning in people’s lives. If community relations, and in turn their relationship with a forest, are valued, that is part of a sustainable society, and provides a platform for continuation and adaptation.

## Electronic supplementary material

Below is the link to the electronic supplementary material.

Supplementary material 1 (PDF 222 kb)
