# Supplementary material for: Forests in common: Learning from diversity of community forest arrangements in Europe
Source: Ambio. 2020 Sep 13;50(2):448–64. doi: 10.1007/s13280-020-01377-x (PMC7782764; doi:10.1007/s13280-020-01377-x)

Ambio

Electronic Supplementary Material
This supplementary material has not been peer reviewed.

Forests in common: Learning from diversity of community forest arrangements in Europe

Anna Lawrence, Paola Gatto, Nevenka Bogataj, Gun Lidestav

Appendix S1. Frequencies of each descriptor under each sub-dimension

These bar charts show the distribution of descriptors for each sub-dimension in the fifteen cases included in our purposive sample. In each bar chart, the horizontal axis reports the number of cases recorded for each descriptor; the vertical axis reports the range of descriptors agreed for each sub-dimension.

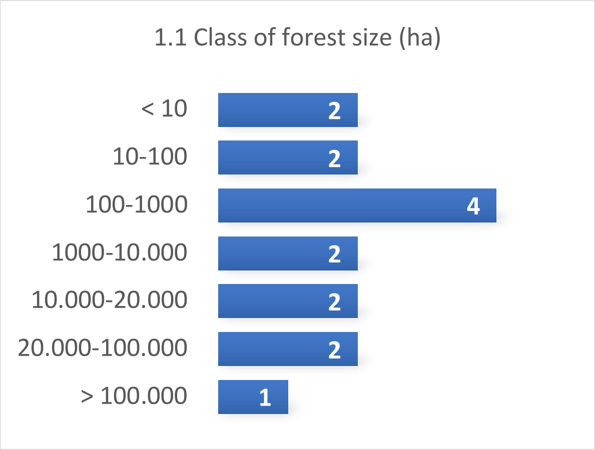

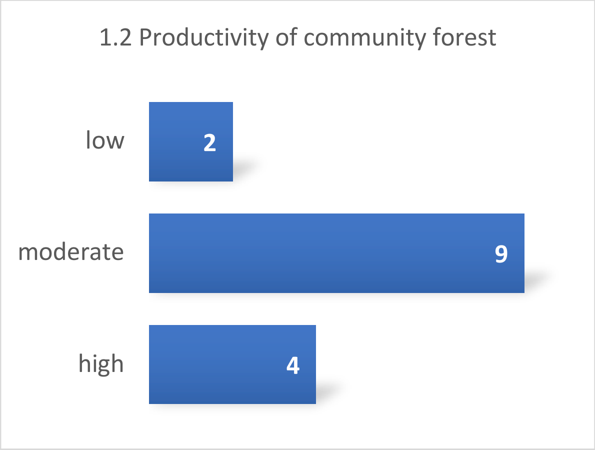

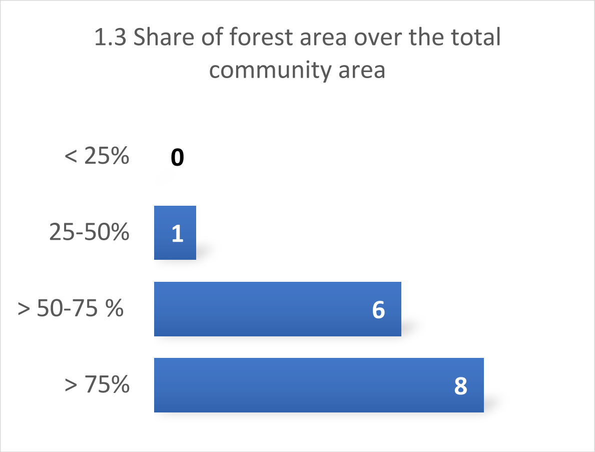

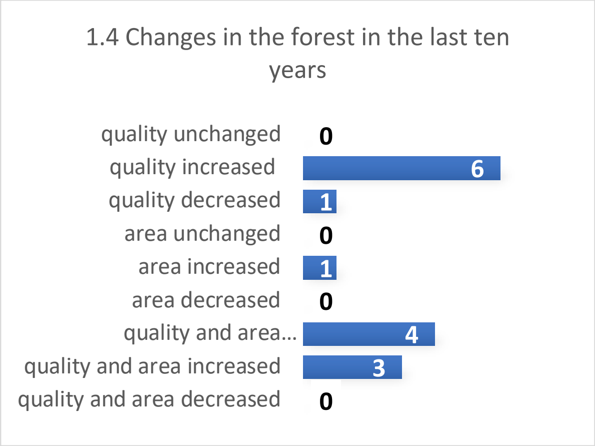

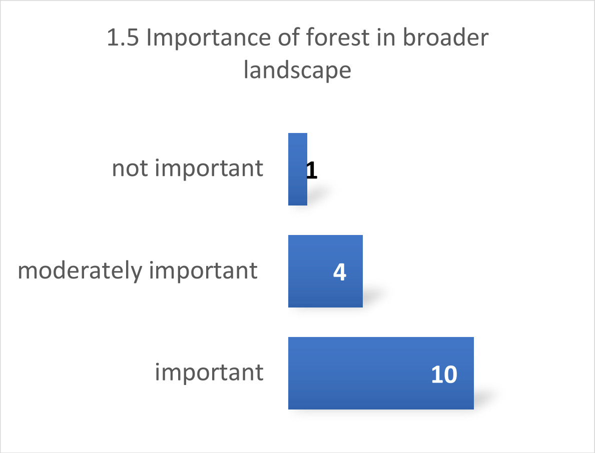

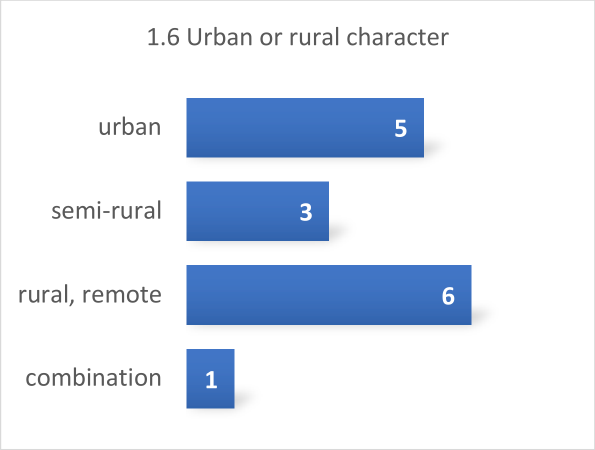

Community characteristics in the selected cases

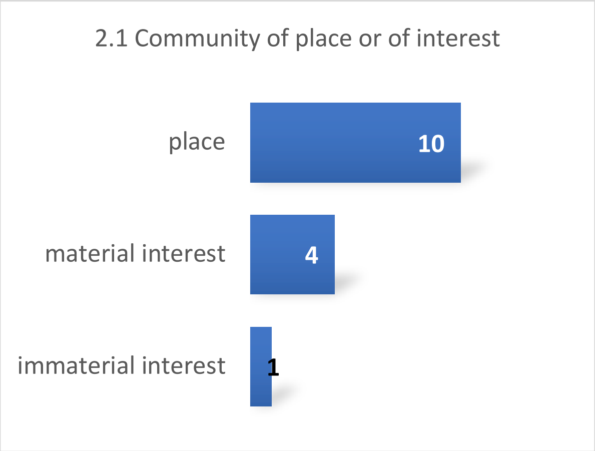

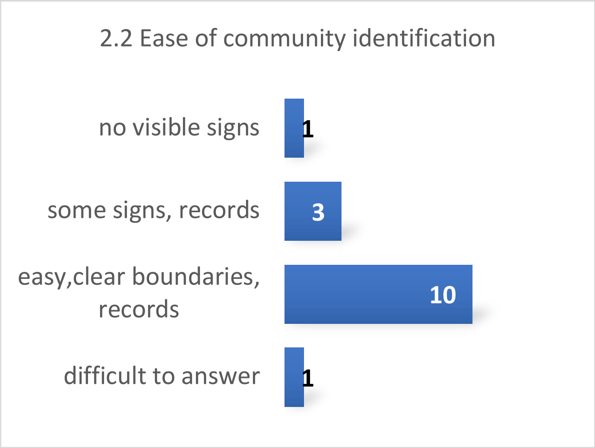

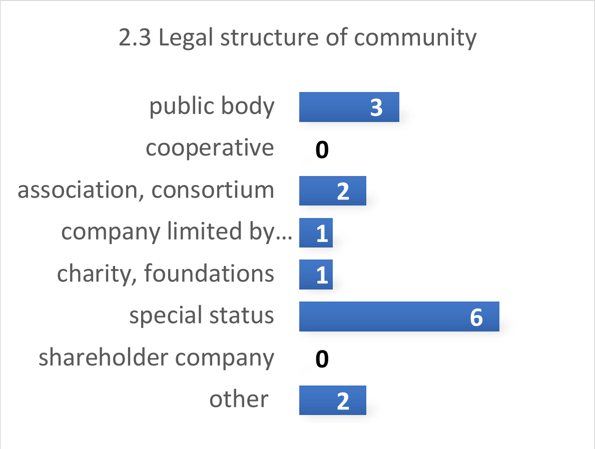

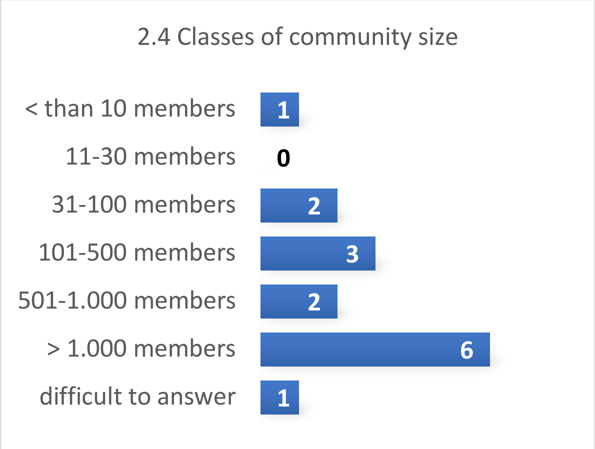

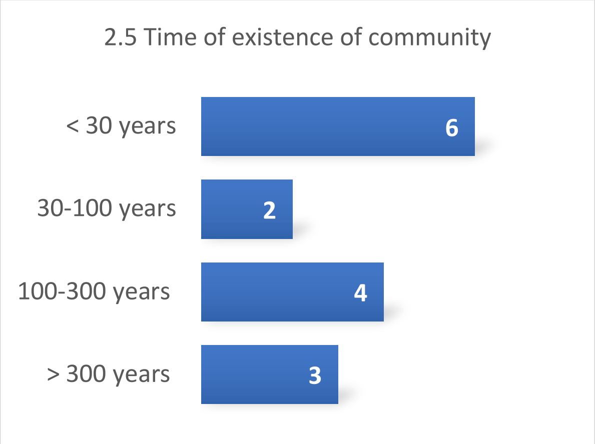

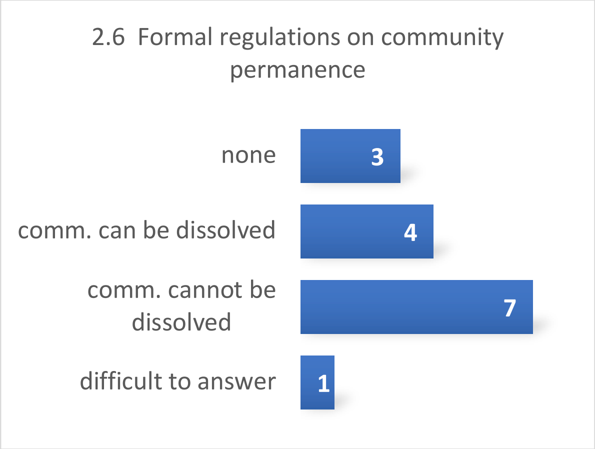

Community characteristics in the selected cases (continued)

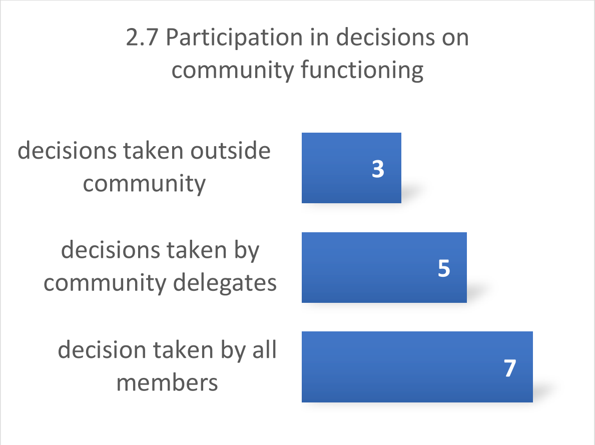

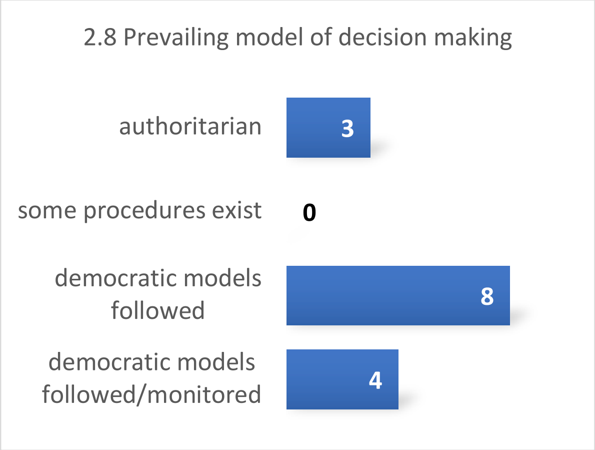

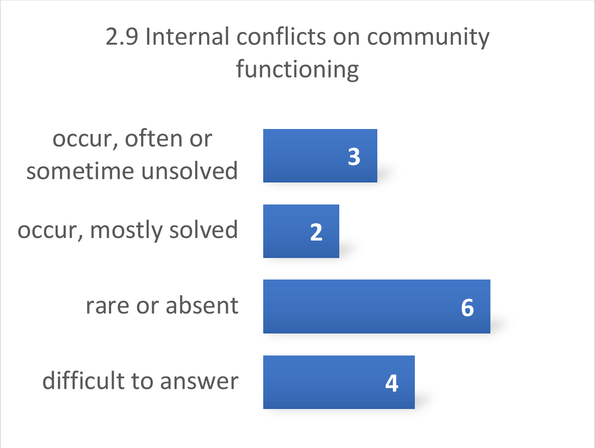

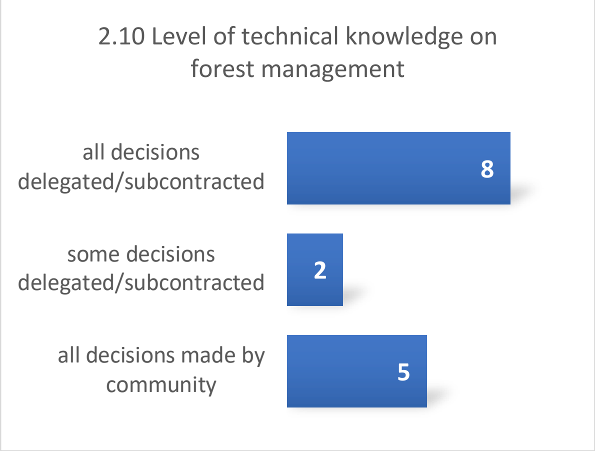

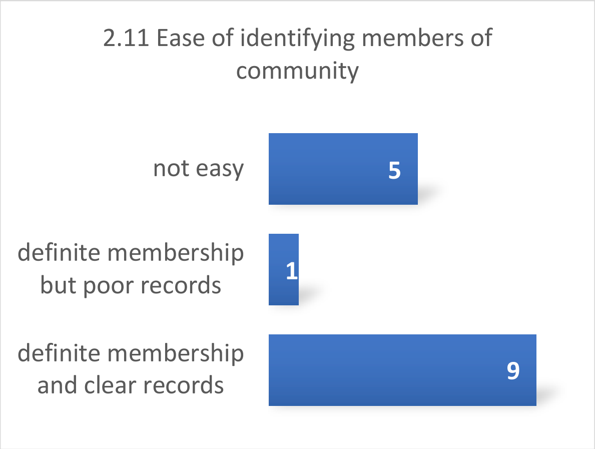

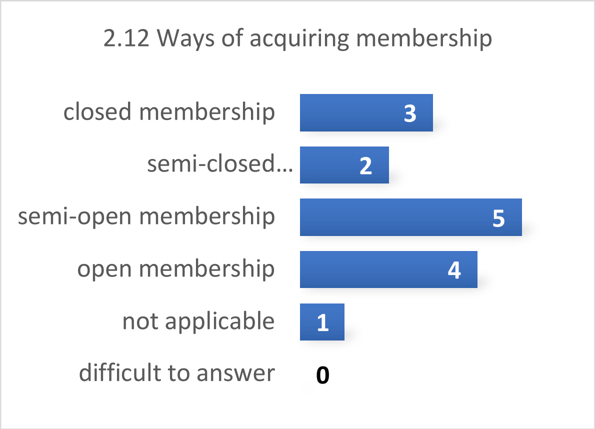

Community characteristics in the selected cases (continued)

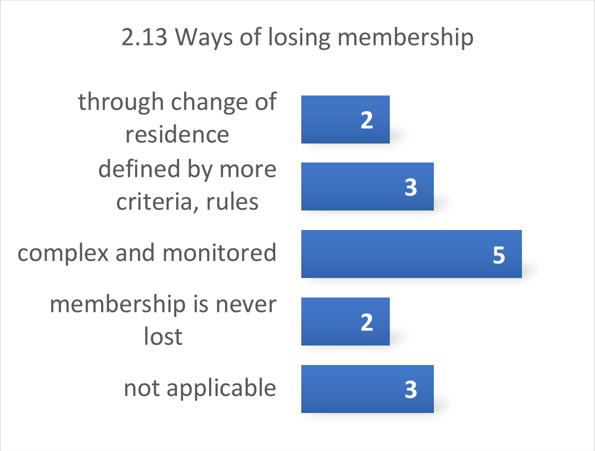

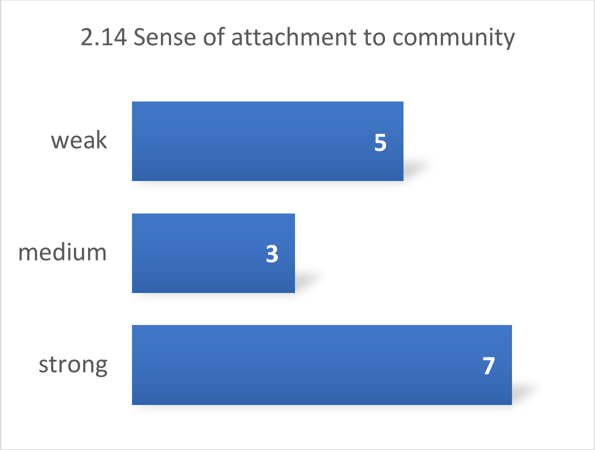

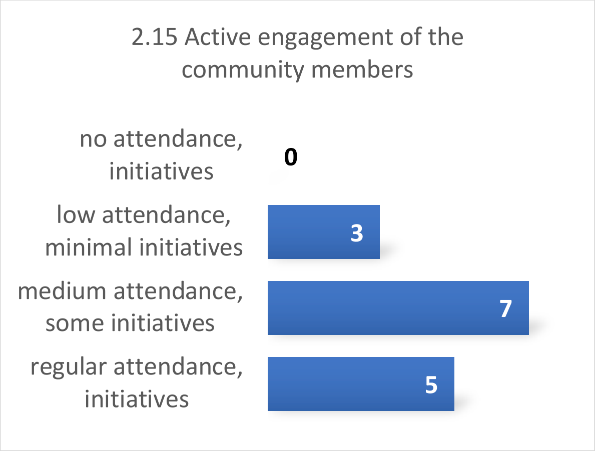

Relationship between community and its forest in the selected cases

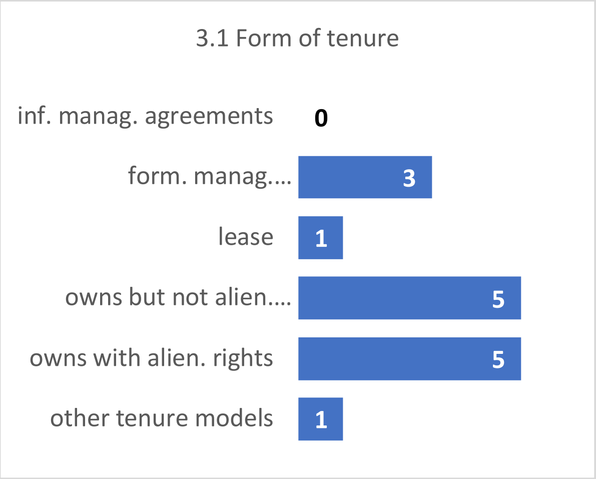

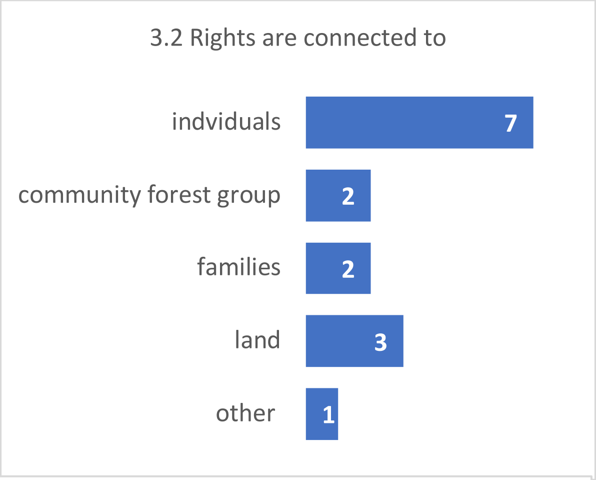

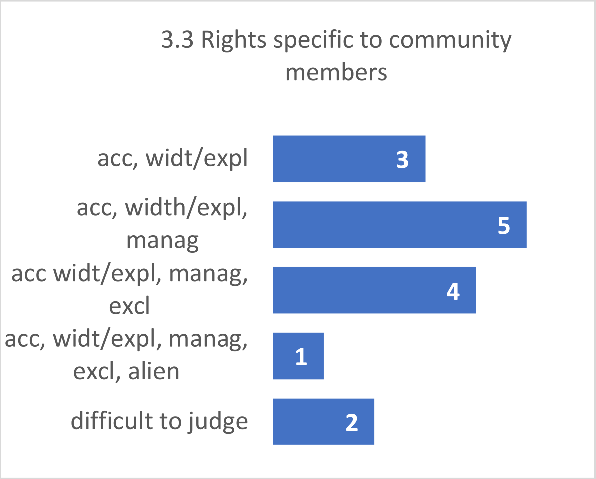

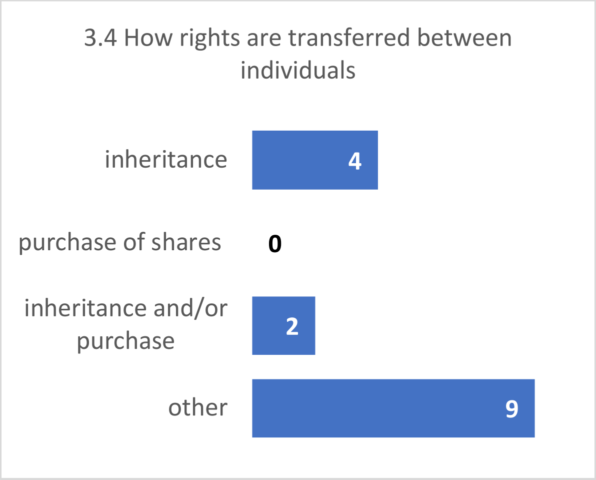

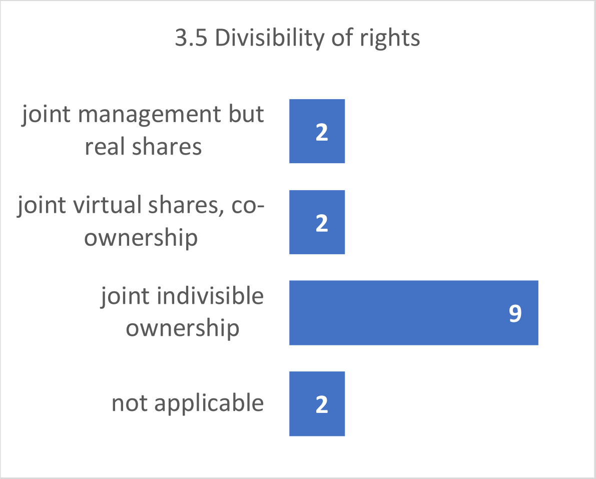

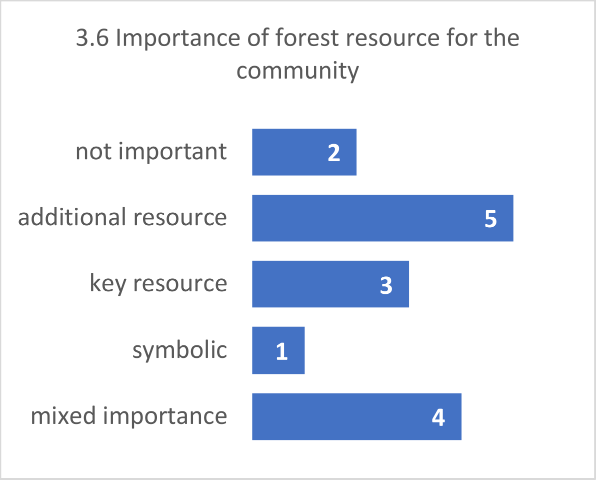

Relationship between community and its forest in the selected cases (continued)

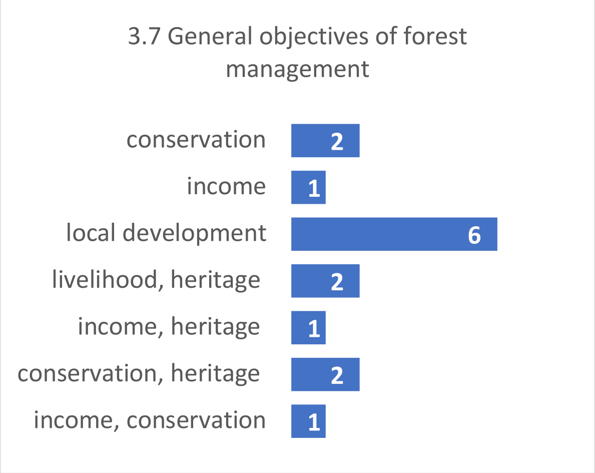

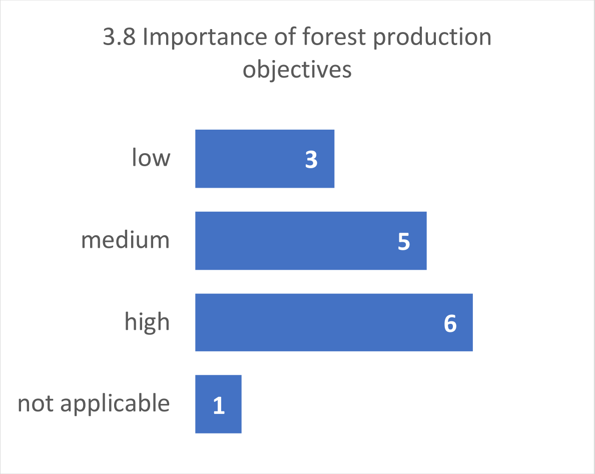

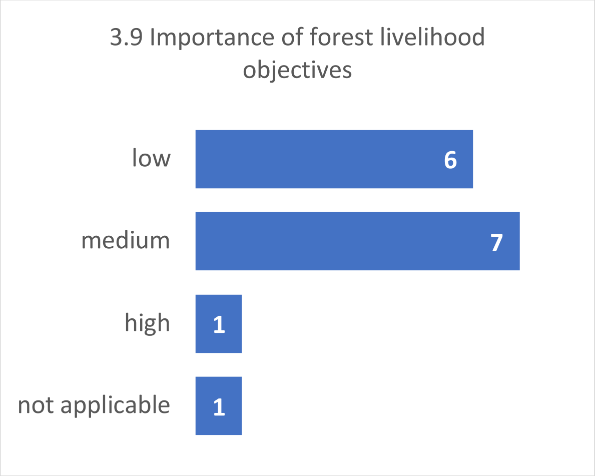

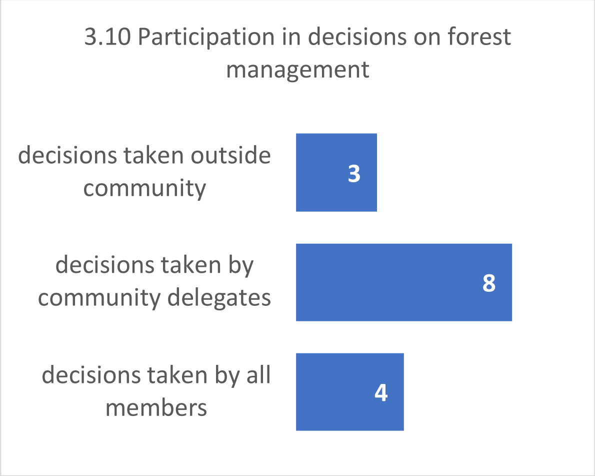

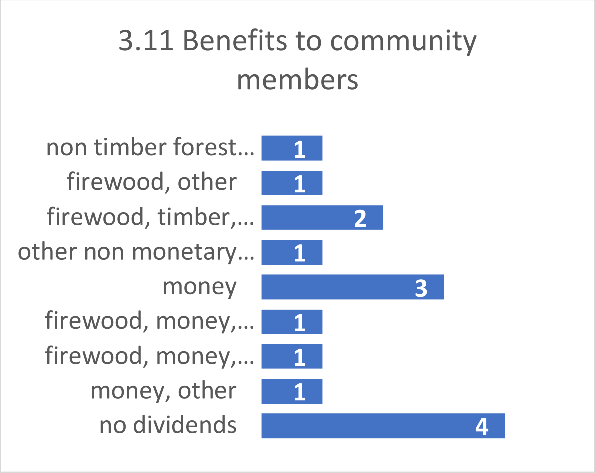

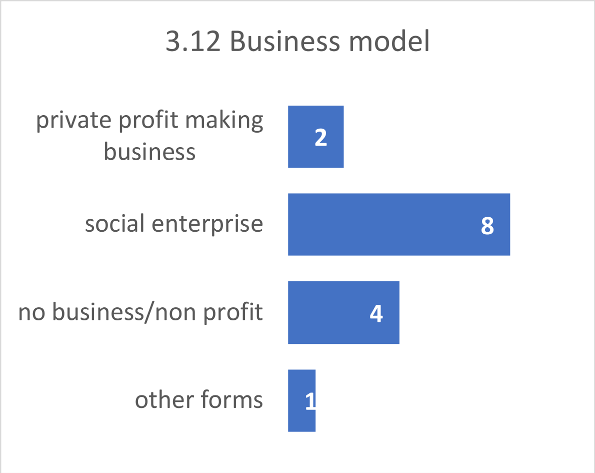

Relationship between community and its forest in the selected cases (continued)

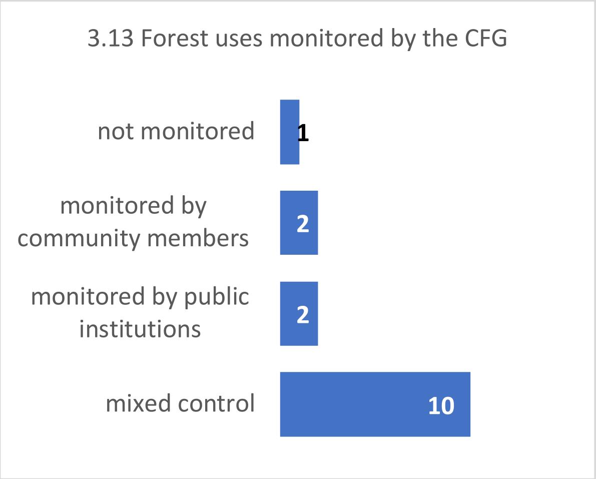

Relationship between community, forest and society in the selected cases

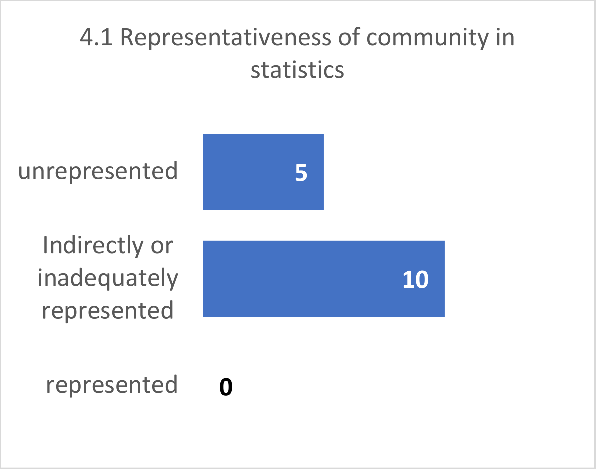

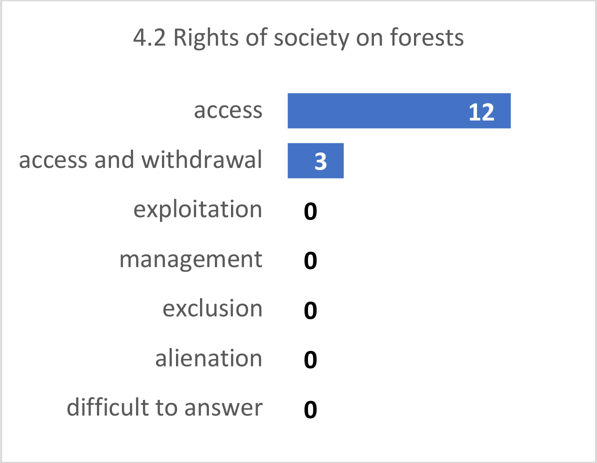

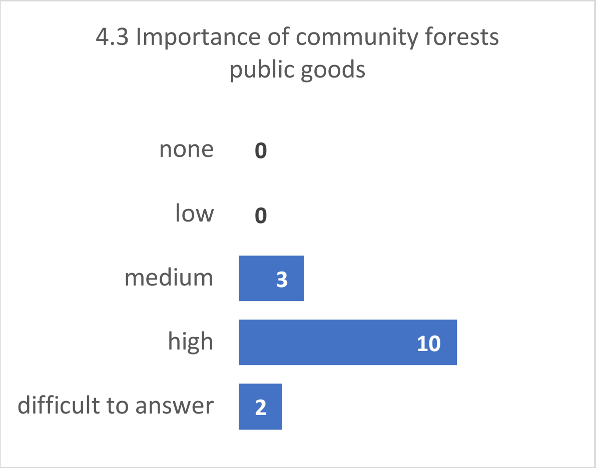

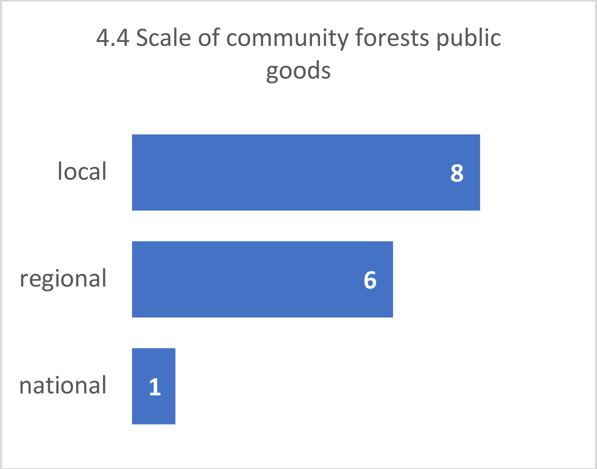

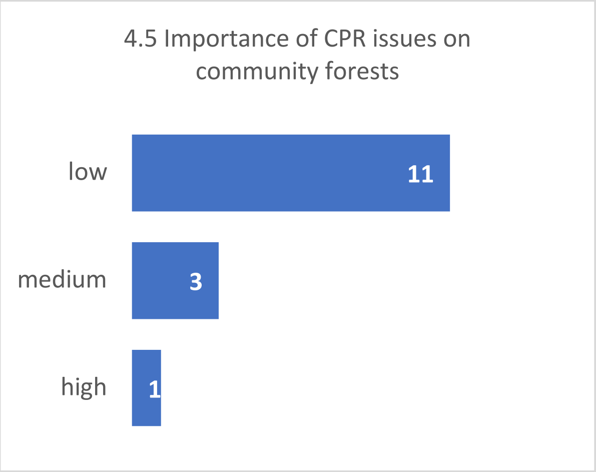

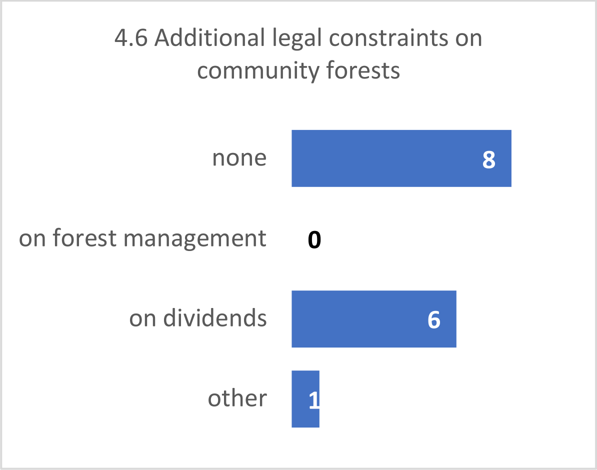

Relationship between community, forest and society in the selected cases (continued)

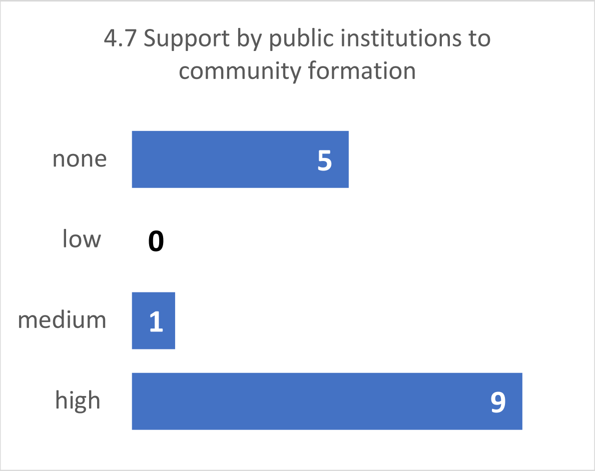

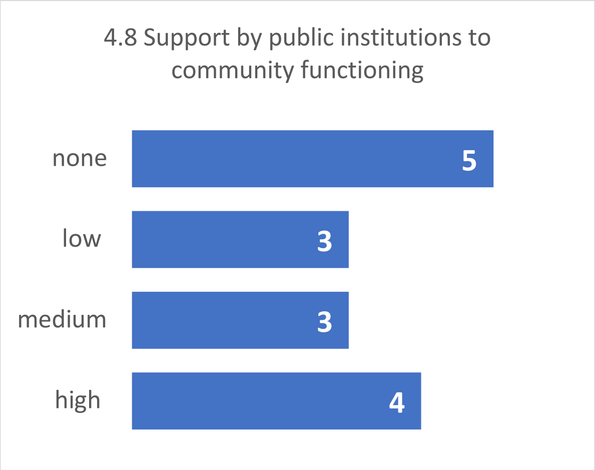

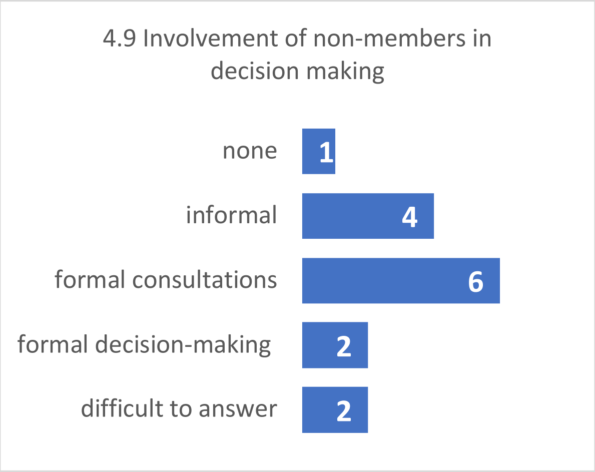

Supplement: Supplementary file 1 — Supplementary material 1 (PDF 222 kb) [file 13280_2020_1377_MOESM1_ESM.pdf]
